# Effect of PET Micro/Nanoplastics on Model Freshwater Zooplankton

**DOI:** 10.3390/polym17091256

**Published:** 2025-05-05

**Authors:** Natan Rajtar, Małgorzata Starek, Lorenzo Vincenti, Monika Dąbrowska, Marek Romek, Rosaria Rinaldi, Francesca Lionetto, Mariusz Kepczynski

**Affiliations:** 1 Faculty of Chemistry, Jagiellonian University, Gronostajowa 2, 30-387 Kraków, Poland; natan.rajtar@doctoral.uj.edu.pl; 2 Doctoral School of Exact and Natural Sciences, Jagiellonian University, Prof. S. Łojasiewicza 11, 30-348 Krakow, Poland; 3 Department of Inorganic Chemistry and Pharmaceutical Analytics, Faculty of Pharmacy, Jagiellonian University Medical College, 9 Medyczna St, 30-688 Kraków, Poland; m.starek@uj.edu.pl (M.S.); monika.1.dabrowska@uj.edu.pl (M.D.); 4 Department of Mathematics and Physics “Ennio De Giorgi”, University of Salento, Via Monteroni, 73100 Lecce, Italy; lorenzo.vincenti@unisalento.it (L.V.); ross.rinaldi@unisalento.it (R.R.); 5 Institute for Microelectronics and Microsystems (IMM), CNR, Via Monteroni, 73100 Lecce, Italy; 6 Department of Cell Biology and Imaging, Institute of Zoology and Biomedical Research, Jagiellonian University, 9 Gronostajowa Street, 30-387 Kraków, Poland; marek.romek@uj.edu.pl; 7 Department of Engineering for Innovation, University of Salento, Via Arnesano, 73100 Lecce, Italy

**Keywords:** nanoplastics, PET, zooplankton, ecotoxicity, Daphnia magna, Thamnocephalus platyurus, ball milling

## Abstract

Micro- and nanoplastic pollutants are among the major environmental challenges, and are exacerbated by the continuous degradation of growing amounts of plastic debris in the aquatic environment. The purpose of this study was to investigate the morphology of micro/nanoplastics (M/NPs) formed from polyethylene terephthalate (PET) by mechanical degradation in an aquatic environment, which mimics the processes in the natural environment well, and to determine the impact of these particles on model aquatic organisms. To this end, M/NPs were obtained by ball milling in an aqueous medium and the effect of milling length on particle size and shape was investigated. The particles obtained in an environment simulating natural conditions were irregularly shaped, and those of nanometric size tended to form aggregates of various shapes. The ingestion and toxicity of PET M/NPs to freshwater zooplankton were then assessed. *Daphnia magna* and *Thamnocephalus platyurus* were used in a series of acute ecotoxicity tests, by exposure to M/NP dispersions at environmentally realistic concentrations (0.01–1.0 mg/L), as well as at very high concentrations (100–1000 mg/L). A significant uptake of PET particles by both types of invertebrates was observed, and the M/NPs were mainly concentrated in the digestive tracts of the crustaceans. However, they did not cause acute toxicity to the tested organisms or a reduction in their swimming activity, even at concentrations as high as 1000 mg/L.

## 1. Introduction

Poly(ethylene terephthalate) (PET, Figure S1) is one of the most widely used plastics in industry. In 2022 alone, 30 million tons of PET were produced globally, of which 40% was food and beverage packaging (including the well-known PET bottles), 24% films and sheets, 18% fibers, 10% consumer goods and 8% other products [1]. In addition, it is used in bioengineering to produce, for example, scaffolds and meshes for in vivo applications [2,3]. Only about 2 million tons of PET waste is recycled each year (a report based on 2022 data) [4]. The remaining plastic produced ends up in landfill sites, from where it is transferred to the environment. Plastic waste then undergoes gradual mechanical and/or photochemical degradation, breaking down into increasingly smaller fragments referred to as micro- and nanoplastics (M/NPs). Analyses of marine coastal and freshwater sediments collected from various sites in South Korea, Japan and the United States revealed the presence of PET MPs at concentrations (dry weight) of up to 13,000 µg/g, 5.4 µg/g and 10 µg/g, respectively [5]. In contrast, a study from 2023 of the state of microplastic contamination on beaches in Malaysia found an alarming amount of MPs in the sediment with a total mass concentration of up to 57.72 mg/g, of which about 11–13% was PET particles [6]. Microparticles can be directly ingested by fish and larger aquatic organisms, while nanoparticles can be filtered by zooplankton and transferred through food chain to higher trophic level organisms [7]. Thus, they enter the digestive systems of organisms that feed on aquatic animals.

The negative effects of micro/nanoplastics (M/NPs) on the environment and human health are still under discussion. Due to the difficulties in sampling M/NPs from the environment, they are artificially produced in the laboratory and their deleterious impact studied in vitro against various cell lines, mostly human [8,9,10,11], and in vivo against zebrafish. In vivo studies with the use of developing zebrafish embryos as an animal model demonstrated the size-dependent distribution and the size- and concentration-dependent toxicity of PET NPs in terms of hatching rate, heart rate, and ROS generation [12]. The toxicity mechanism of PET NPs in intact zebrafish embryos was explored in detail by Bashirova et al. [13]. PET NPs were shown to cause significant alterations in hatching and survival rates of the embryos.

Environmental cytotoxicity tests are a natural extension of research on the interaction of nanoplastics with living organisms. Since most nanoplastics are found in water, tests were performed on fresh- and saltwater organisms such as zooplankton. However, the effects of PET micro- and nanoplastics on zooplankton have not yet been studied, despite the fact that large amounts of this plastic are present as contaminants in standing water bodies and that their potential as carriers of pollutants in the aquatic environment has been demonstrated [14]. As with the cytotoxicity of nanoplastics, most studies with zooplankton have used polystyrene (PS) as model nanoparticles. Due to the transparency of its body, *Daphnia magna* (*D. magna*) is one of the most commonly used model organisms for ecotoxicity testing [15]. For example, positively modified PS NPs were shown to exhibit acute toxicity against *D. magna*, and particles of smaller size had a stronger effect [16]. The toxicological mechanism of various functionalized polystyrene (PS) NPs on this invertebrate was studied by Lin et al. [17]. It was shown that the functionalized PS NPs were less toxic than regular PS. De Felice et al. studied the effects induced by long-term exposure (21 days) to two sublethal concentrations of PS NPs (0.05 and 0.5 μg/mL) on D. magna [18]. They showed that such low concentrations of nanoparticles pose a limited risk to aquatic organisms. *D. magna* was used as a model zooplankton in the study of the effects of lifetime exposure (102 days) to sub-lethal concentrations of positively and negatively modified PS NPs [19]. The effects of PS particle size, exposure time and the presence of food on the ingestion and excretion of micro- and nanoplastics by *D. magna* were investigated [20]. Ecotoxicity assays were also conducted on other model organisms, including *Thamnocephalus platyurus* (*T. platyurus*) larvae or *Brachionus calyciflorus* rotifers [21]. The research showed that PS nanoparticles exerted different toxic effects depending on the type of freshwater model organism used. Of the three invertebrates tested, *T. platyurus*, *Brachionus calyciflorus*, and *T. platyurus*, the latter was the most sensitive to the presence of plastic contaminants [21].

The aim of this study was to investigate the detrimental effects of PET-M/NPs on freshwater zooplankton. For this purpose, we first focused on several issues regarding the production of micro-/nanoplastics from PET waste. In our work, we attempted to replicate as closely as possible the plastic particles that can be found in the environment, obtaining different shapes and sizes. Mechanical milling mimics the degradation process of floating plastics in the natural environment well due to mechanical abrasion occurring in the mechanical breakdown process of plastic, for example, on the beaches of open water reservoirs [22,23]. This method leads to the preparation of mixtures of NPs and MPs that are present in real environmental systems (plastic particles of different sizes can simultaneously affect aquatic organisms). Therefore, we selected the ball milling method as the one that most closely resembles the mechanical degradation process of plastic waste occurring in the environment. The morphology and size of the as-prepared PET particles were characterized by microscopic methods. Their stability in an aqueous medium and the possibility of aggregate formation were also analyzed. The effects of M/NPs on freshwater organisms were then analyzed using *D. magna* and *T. platyurus.* These organisms, which are major consumers in freshwaters ecosystems, are sensitive to various pollutants and are commonly used in ecotoxicological studies [24]. Using fluorescently labeled PET particles, their ingestion by these two model invertebrates was determined. Finally, the survival of the freshwater organisms in the presence of PET M/NPs in a wide range of concentrations was investigated by performing acute toxicity tests.

## 2. Materials and Methods

### 2.1. Materials

Polyethylene terephthalate (PET) pellets (commercial name RT52), with a cylindrical shape, approximately 4 mm in length and 2.4 mm in diameter, with a smooth surface typical of industrial polymer granules [25] and an intrinsic viscosity of 0.634–0.638 dL/g, were obtained from Invista Resins & Fibers GmbH (Gersthofen, Germany). The molecular weight of the PET chains was estimated using the Mark–Houwink equation

(1) $$\left[\eta \right]=KM_{w}^{a}$$

where [*η*] is the intrinsic viscosity, *M_w_* is the average molecular weight, and *K* and *a* are constants. Using the *K* and *a* values reported in the literature for PET [26,27], an average molecular weight of 29,500–46,000 g/mol was obtained.

Nile red (NR, dye for microscopy) and acetone were purchased from Sigma-Aldrich (St. Louis, MI, USA) and used as received. All experiments were performed in Milli-Q water.

### 2.2. Preparation of PET Micro/Nanoplastics (PET-M/NPs)

PET pellets were ground using a S/1 1000B ball mill (Ceramic Instruments S.r.l, Sassuolo Modena, Italy) equipped with a zirconia (ZrO_2_) jar (0.3 L) and operating at 390 rpm in a two-step procedure. In the first step, the pellets (1 g) were ground using 10 mm zirconia balls (100 g suspended in 100 mL of water) for 30 cycles, each lasting 2 min with a 5 min break. Every 10 cycles, the jar was placed in a –80 °C freezer for 8 min. To remove the zirconia balls, the suspension was sieved through a 150 μm sieve. In the second step, 500-μm zirconia balls (10 g) were added to the PET suspension (60 mL) and milled for 10, 15, or 20 cycles, each lasting 2 min with a 5 min break. After milling, the suspension was passed through 150 μm sieves to remove the zirconia balls. Changes in PET particle size during the grinding cycles were tracked by laser diffraction. PET-M/NPs were recovered by freeze-drying (–51 °C and 0.48 mbar).

For fluorescence staining of the micro/nanoparticles, NR (1 mg) was dissolved in acetone (1 mL) and added to water (10 mL). PET-M/NPs (20 mg) were mixed with the NR solution (3 mL) and left for 24 h in the dark. To remove unbound dye, the dispersion was dialyzed into water (1 L) for three days in the dark. The water was changed after 30 min, 1 h, and 2 h, and then twice a day. Fluorescently labeled PET particles (NR_PET-M/NPs) were recovered by freeze-drying (–51 °C and 0.48 mbar).

### 2.3. Laser Diffraction (LD) and Zeta Potential Measurements

The analysis of particle size distribution was carried out using a CILAS 1190 multi-laser particle size analyzer and Particles Expert software v.9.5 CPS Us, Inc., Madison, WI, USA), as described previously [28]. Sample volume (1 to 5 mL) was adjusted based on particle concentration to obtain the right obscuration for a good measurement, that is, the percentage of the laser beam that is scattered by the sample particles.

Zeta potential measurements were performed using a Malvern Nano ZS instrument (Worcestershire, UK) with a light scattering angle of 173° as previously described [29]. The results were analyzed using the software provided by the manufacturer.

### 2.4. Transmission Electron Microscopy (TEM)

Specimens were prepared by placing a drop (~5 μL) of the micro/nanoparticle suspension on a TEM grid (Formvar/Carbon Supported Copper Grids, grid size 300 mesh, Sigma-Aldrich). The grids were allowed to settle for 5 min, and the excess sample was blotted with filter paper and dried at room temperature. The specimens were imaged under a JEOL JEM-2100HT microscope (Jeol Ltd., Tokyo, Japan) operating at an accelerating voltage of 80 kV and set to 15,000× magnification. Bright-field TEM images were captured using a 4K × 4K CCD camera CMOS camera (TVIPS—Tietz Video and Image Processing Systems, Gauting, Germany) equipped with EMMENU4 software ver. 4.0.9.87.

### 2.5. Atomic Force Microscopy (AFM)

Briefly, 10 μL of M/NP dispersion was dropped onto freshly cleaved mica and allowed to dry at room temperature for 60 min. AFM images were acquired using an AFM NTEGRA instrument (NT-MDT Spectrum Instruments, Moscow, Russia), equipped with CSG01 tips and operating in contact mode. In detail, the CSG01 probes are characterized by a tip radius of curvature of 10 nm and a nominal force constant ranging from 1.45 N/m to 15.1 N/m. IA-P9 v. 3.4 software was used for AFM data analysis and visualization. Grain analysis was performed on the AFM topography images by a specific tool of IA-P9 v. 3.4 software.

### 2.6. Toxicity of PET Micro/Nanoplastics to Aquatic Ecosystem Organisms

To investigate the toxicity of PET micro/nanoparticles towards freshwater organisms *T. platyurus* and *D. magna* two commercially available microbioassays were used: Thamnotoxkit F and Daphtoxkit F kits (MicroBioTests Inc., Gent, Belgium), respectively.

#### 2.6.1. Thamnotoxkit F

The 24 h acute toxicity bioassay was based on instar II-III larvae of the fairy shrimp *T. platyurus* that hatched from cysts. Tests were conducted according to the manufacturer’s instructions [30]. Standard freshwater containing NaHCO_3_ (96 mg/L), CaSO_4_ (120 mg/L), MgSO_4_ (123 mg/L), and KCl (4 mg/L), which corresponded to moderately hard water, was prepared. The tests were carried out in 24-well plates for 5 concentrations of PET-M/NPs (0.1, 1, 10, 100, and 1000 mg/L, 1.5 mL) in the standard freshwater, in 3 replicates. The larvae obtained by 24 h hatching of the cysts were transferred to separate wells with dispersions, with approximately 10 larvae per well, and incubated at 25 °C for 24 h in the dark. After this time, the number of live and dead larvae was counted under a microscope.

#### 2.6.2. Daphtoxkit F

The bioassay used the neonates of *D. magna* crustaceans that were hatched from ephippia. The acute toxicity microbiotest was performed in accordance with the manufacturer’s instructions [31]. Standard freshwater was prepared with a composition of NaHCO_3_ (64.75 mg/L), CaCl_2_ (294.00 mg/L), MgSO_4_ (123.25 mg/L), and KCl (5.75 mg/L), which corresponded to the natural freshwater recommended by ISO6341 for the acute toxicity test with *D. magna*. The test began with the hatching of the neonates from dormant eggs. For this purpose, ephippia were transferred to a microsieve, rinsed with tapwater, and then placed in a Petri dish with standard freshwater. The tests were carried out in 24-well plates for 5 concentrations of PET-M/NPs (0.1, 1, 10, 100, and 1000 mg/L, 1.5 mL) in the standard freshwater, in 4 replicates. Five neonates were placed in each well with dispersion. The plates were incubated at 20 °C in the dark. After 24 and 48 h, the number of dead and immobile organisms was compared with the number of actively swimming organisms.

### 2.7. Confocal Microscopy Analysis

To assess the ingestion of M/NPs by *D. magna* and *T. platyurus*, crustaceans (<24 h old) were exposed to NR_PET-M/NPs at a concentration of 100 mg/L for 24 h for *T. platyurus* and 48 h in the case of *D. magna*. At the end of the exposure, at least 3 individuals were transferred to a drop of pure water placed on a microscope slide. The samples were observed under an A1-Si Nikon Inc. (Tokyo, Japan) confocal laser scanning system (LSCM) built onto a Nikon inverted microscope Ti-E using a 4× objective. Images were acquired at a resolution of 2048 × 2048. The Al−Si system was equipped with a 488 nm diode laser and LSBF imaging by the diascopic detection of forward-scattered excitation laser light during confocal laser scanning.

## 3. Results and Discussion

### 3.1. Preparation of PET Micro/Nanoplastics

**Figure 1:**
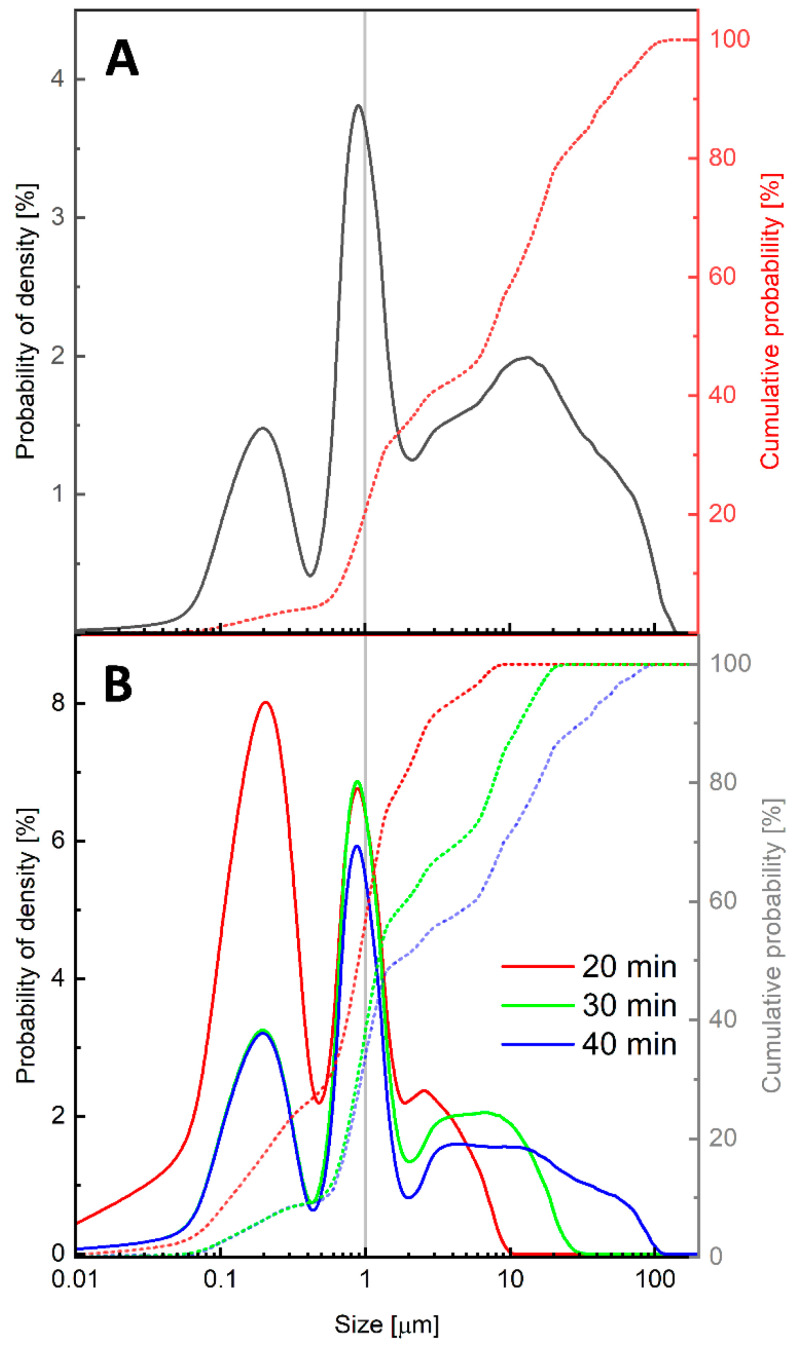
Size distributions obtained by laser diffraction measurements for PET particles after milling with 10 mm zirconia balls for 60 min (**A**) and with 500 μm zirconia balls for 20, 30, and 40 min (**B**). The solid lines show the probability of density (referring to the left ordinate axis) and the dotted lines show the cumulative probability (referring to the right ordinate axis). The gray vertical lines indicate a particle diameter of 1 µm.

In this work, a two-step ball milling protocol was used to produce model PET particles. This technique allowed for the preparation of nanoparticles without the use of toxic solvents and the simulation of the formation of such particles in a natural aquatic environment due to the mechanical degradation of plastic waste. The size of the polymer particles was reduced to the micro/nanometer scale as a result of friction between the polymer material and the zirconia beads. The size distribution of the PET particles formed during the milling process was monitored using laser diffraction. The probability of density curves and the cumulative probability curves are reported in Figure 1. The density probability curves show the relative amount of particles in each particle size range. The cumulative probability curve shows the total percentage of particles that are smaller than a given size. In the first step, the PET pellets were ground for 60 min using 10 mm zirconia balls. The as-prepared particles were then sieved through sieves with a pore size of 150 μm. The size distribution showed that about 20% by volume of the PET particles were smaller than 1 μm (Figure 1A). In the second milling step, 500 μm zirconia beads were used, which significantly increased the number of particles with sizes below 1 μm. After 20 min of grinding, about 60% of the particles had sizes in the nanometer range. Interestingly, increasing the milling time resulted in a gradual increase in particle size (Figure 1B). This was probably due to the merging of smaller particles into aggregates of large sizes. In further studies, we used the PET-M/NPs obtained after 20 min of milling with 500 μm zirconia beads.

The surface potential is considered to have a significant effect on the stability of colloid systems. Particle dispersions characterized by ζ-potential values below −30 mV or above +30 mV are generally considered stable, which is the result of strong repulsion between particles that prevents their aggregation [29]. The zeta potential measured for the PET-M/NP dispersion was –22.6 ± 0.9 mV. This indicates that the repulsive forces acting between the PET particles may not be sufficient to prevent their aggregation. Zhang et al. investigated the changes in zeta potential for PET particles at different pH levels [32]. At pH 7, the zeta potential was about –10 mV, which is higher than the value obtained in our measurements. The difference may be due to different measurement conditions, in particular different ionic strength. It was shown that the zeta potential of a particle depends strongly not only on the pH, but also on the ionic strength of the solution [33]. Dong et al. showed that the aggregation kinetics of PET nanoplastics increased with increasing ionic strength and decreasing solution pH [34]. Nevertheless, the zeta potentials measured in this work, as well as those reported in the literature, clearly indicate the low stability of the PET-M/NP dispersion.

**Figure 2:**
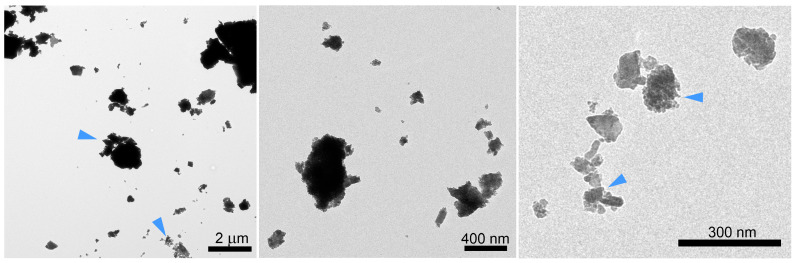
Typical TEM pictures of PET micro/nanoplastics prepared by ball milling, showing the morphologies at three different scale sizes. Blue arrowheads indicate aggregates of PET nanoparticles.

The morphology and size of the PET-M/NPs obtained by ball milling were examined with AFM and TEM microscopes. Figure 2 shows typical TEM micrographs of the particles. The PET-M/NPs were characterized by irregular shapes with a wide diameter distribution extending from tens of nanometers to a few micrometers. In addition, TEM images highlighted the presence of aggregates, consisting of nanometer-scale particles. Microscopic analysis showed that the ball milling procedure allowed the preparation of nanoplastics from PET waste, but the obtained nanometer-sized particles aggregate to form larger clusters, which is in agreement with the results of the laser diffraction measurements.

**Figure 3:**
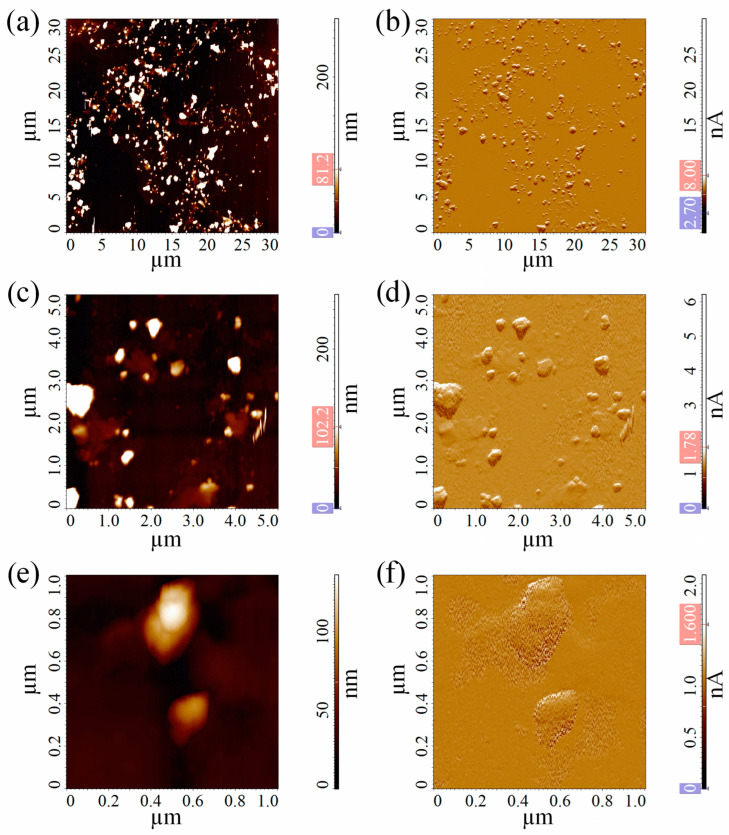
Height (**a**,**c**,**e**) and deflection (DFL) (**b**,**d**,**f**) images from contact mode AFM measurements in air of PET micro/nanoparticles prepared by ball milling and deposited on the mica surface. The samples were scanned at three different scale sizes: 30 μm × 30 μm (**a**,**b**), 5 μm × 5 μm (**c**,**d**), and 1 μm × 1 μm (**e**,**f**).

To further analyze the morphology and size of the PET-M/NPs, we performed AFM measurements. Figure 3 presents typical AFM images taken for the PET micro/nanoparticles prepared by ball milling, which were deposited on the mica surface. The images show polydisperse irregular objects. The size heterogeneity of the PET nanoparticles was confirmed by grain analysis of the 30 μm × 30 μm areas with the exclusion of objects with an area smaller than 0.003 μm^2^ due to resolution reasons (Figure 3a,b), which was representative of the sample morphology. The size histogram from the grain analysis, shown in Figure S3, indicates that PET particles are mainly in the tens to hundreds of nanometers size range. To better analyze the morphologies of individual nanoparticles, the samples were scanned in smaller areas (5 μm × 5 μm and 1 μm × 1 μm), and their topography is presented in Figure 3c,e. The DFL signals associated with these topography images (Figure 3b,d,f) were relevant to better visualize the boundaries and the nanofeatures of individual nanoparticles. The nanoparticles did not show a unique shape; objects with both round and very non-spherical shapes were observed, the latter characterized by irregular edges.

Several methods have been proposed to produce model nanoparticles from PET material for studying their interaction with biological systems. Magrì et al. used laser ablation to form PET nanoplastics with controlled size distribution from polymer films [8]. As shown by TEM analysis, the NPs prepared by laser ablation had an approximately spherical shape, although some clusters were observed in the as-synthesized samples, which were considered typical of real nanoplastic samples. However, laser ablation has a very low yield. Another proposed method for producing PET nanoparticles involves precipitating polymer particles from a polymer solution dissolved in a concentrated solution of trifluoroacetic acid [11] or hexafluoroisopropanol [10]. These methods make it possible to produce large quantities of PET micro/nanoparticles. However, they require the use of toxic solvents, which must be removed in an additional procedure. In addition, the particles obtained in this way are also characterized by a spherical morphology. Micrometer-sized PET flakes of various sizes were obtained by grinding 1 mm diameter granules using industrial mills [35,36]. On the other hand, Ji et al. [12] and Caldwell et al. [9] used mechanical milling to produce PET nanoparticles. The procedures described included pre-milling, followed by sieving and grounding in a ball mill or centrifugation of particle suspensions stabilized with dispersants. A two-step procedure for grinding PET material was proposed by Lionetto et al. [28] and consisted of initial milling with an ultracentrifugal mill followed by ball milling. Mechanical milling has some limitations related to low efficiency and the long time required to obtain nanometer sizes [35], but the resulting particles are mostly characterized by irregular shapes that more closely resemble the morphology of micro- and nanoplastics formed under real conditions. The shape of the nanoplastic is important in biological research. As shown previously, M/NPs can have different effects on the soil microbiome depending on their shape [37]. In addition, particles with irregular shapes showed stronger responses from primary human monocytes and monocyte-derived dendritic cells compared to spherical ones [38]. Thus, the selection of an appropriate method for the preparation of the plastic material (and thus the morphology of the prepared nanoparticles) can have a direct bearing on the results obtained in ecotoxicity studies.

### 3.2. Effect of PET NPs on Aquatic Organisms

**Figure 4:**
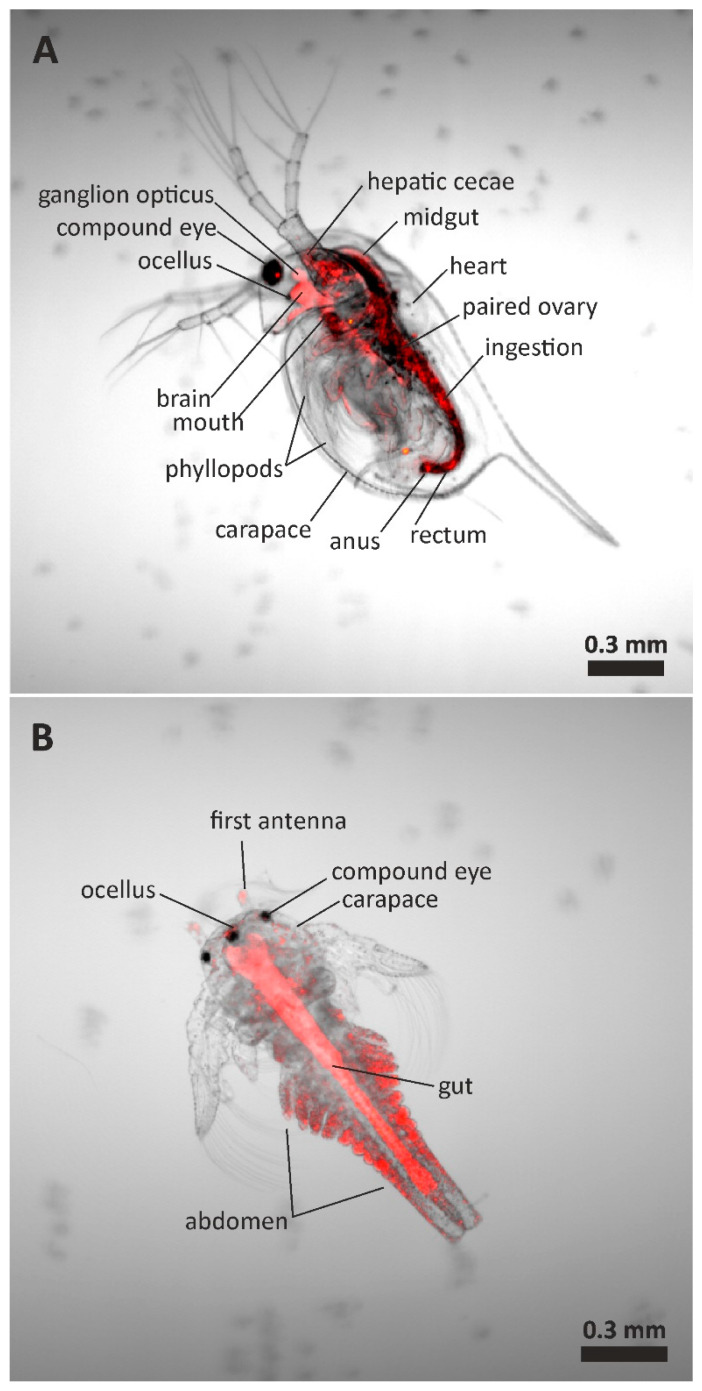
Confocal micrographs showing individuals of *D. magna* (**A**) and *T. platyurus* (**B**) exposed to a 100 mg/L dispersion of fluorescently labeled (Nile red) PET micro/nanoplastics for 24 (*T. platyurus*) and 48 (*D. magna*) hours. Fluorescence from PET-M/NPs (red) was superimposed on the transmitted light images of the crustaceans. The size of *D. magna* and *T. platyurus* individuals is 1.81.4 and mm, respectively.

To evaluate the potential ecological risks of PET-M/NPs, their impact on the viability of freshwater zooplankton was investigated. Two types of crustaceans, *D. magna* and *T. platyurus*, which are present in freshwater reservoirs, were used for the study. Plastic particles, depending on their size, can interact with crustaceans in different ways; they can adsorb on their surface and/or be ingested by them. To understand the interactions between PET-M/NPs and zooplankton, uptake experiments of fluorescently labeled particles were carried out. Such a procedure allowed for the direct visualization of particle ingestion by organisms with transparent bodies, such as the freshwater invertebrates *D. magna* and *T. platyurus*. Therefore, freshly hatched crustaceans were incubated in NR_PET-M/NP dispersions (100 mg/L), and particle ingestion was monitored using confocal microscopy (Figure 4). The microscopic analysis revealed intense red fluorescence, characteristic of NR, localized within the outline of the organisms; however, a clear difference in the distribution pattern of PET particles can be seen between the crustaceans studied (Figure S2). Control organisms did not show any red fluorescence.

Figure 4A depicts a typical image of daphnoids that were incubated for 48 h in the NR_PET-M/NP dispersion. The anatomical structure of *D. magna* has been described in detail in the literature [39], allowing us to describe the different organs in the picture. Fluorescence is mainly observed throughout the digestive system, while less strong fluorescence is localized in the phyllopod area. The presence of fluorescent material in the gut of exposed individuals indicated the effective filtration and uptake of fluorescent M/NPs, and strong fluorescence was still observed in the intestine after a 48 h exposure period. *D. magna* feeds on small suspended particles through efficient water filtration [39]. Food was collected by a filtering apparatus consisting of leaf-like thoracic legs (phyllopods). *D. magna* collect particles that are carried into the food groove by special bristles and typically consumes particles 1–50 μm in diameter [39]. Geller and Muller reported that filter mesh-sizes of *D. magna* are in the size range of 0.24–0.64 μm [40]. Therefore, particles with sizes larger than this range can be actively filtrated. In the case of PS NPs, it was suggested that nanoparticles can still be ingested by passive uptake mechanisms in which small particles interact with and attach to big particles like algal cells or detritus that are actively filtered, or they could be ingested with water while drinking [20]. As we have shown above, PET nanoparticles readily aggregate in the aqueous environment to form clusters of larger sizes that can already be actively captured by the daphnoid filtration apparatus. Interestingly, we did not observe fluorescence localized on the surface of individuals, such as on the carapace. However, adsorption in the compound eye was visible in all cases, showing that this organ of D. magna is exposed to PET-M/NPs.

In the case of *T. platyurus*, red fluorescence was observed throughout the digestive system after only 24 h of exposure (Figure 4B). The presence of NR_PET in the gut of exposed individuals indicated a rapid uptake of PET particles suspended in the aquatic environment. Unfortunately, we found no description in the literature of the mode of food uptake by these crustaceans. In contrast to *Daphnia*, red fluorescence was also observed on the surface of *T. platyurus* specimens (Figure S2), and particularly visible in the abdomen segments, as well as the carapace and first antenna. This shows that micro- and nanoparticles of PET can adsorb to the surface of the *T. platyurus* organism.

The toxic effects of PET-M/NPs on zooplankton were tested at two concentration ranges; 0.1–10 mg/L, which is considered to reflect realistic environmental concentrations, and at high concentrations of 100–10,000 mg/L (Table 1). The results suggest that the short-term exposure of *D. magna* and *T. platyurus* to PET-M/NPs may not result in immediate lethal effects under the experimental conditions used.

The ecotoxicity of PET nanoplastics has not been studied before, while such studies were conducted for model particles made of PS, for which a relationship was found between their size and surface modification and ecotoxicity. Toxicity testing of PS microparticles and nanoparticles modified with amine groups was conducted on *D. magna.* The tests showed no toxicity after 96 h of incubation even up to a concentration of 400 mg/L, while the survival rate of daphnoids treated with NPs at this concentration dropped below 40% relative to the control after just 48 h [41]. Sublethal effects of PS, polyvinyl chloride (PVC) and polyethylene (PE) nanoparticles were tested on *D. magna* [42]. During 48 h of incubation with a concentration range from 2.5 to 250 μg/L, biochemical (amount of reactive oxygen species and activity of the antioxidant enzyme catalase) and behavioral (swimming behavior) responses were investigated. The results showed that the chemical structure of the polymer of which the nanoparticle is composed has a strong influence on the behavior of daphnoids. In contrast, the exposure of *D. magna* to NPs made from unmodified PS poses a limited risk to the individuals at concentrations of 0.5 mg/L even after a 21-day incubation [15]. Mattsson et al. conducted extensive toxicity studies of PS NPs modified with amino, carboxyl and sulfonic groups [43]. Of the tested nanoparticles of different sizes and charges, only amino-modified positively charged PS NPs with a diameter of about 50 nm had a negative effect on *D. magna*, while larger particles of the same material had no effect on daphnoids. For smaller-diameter particles, toxicity was strongly dependent on their concentration. Up to a concentration of 25 mg/L, all organisms were still alive after 24 h, and above 75 mg/L, all were dead within 13 h. In conclusion, despite some discrepancies, it can be said that the surface charge and size of plastic particles have a major impact on their ecotoxicity. Positively charged and small-sized particles of plastics can significantly reduce zooplankton survival. The PET M/NPs we tested, carrying a negative charge on them, did not show acute toxicity even up to very high concentrations.

## 4. Conclusions

In this work, we performed studies of the interaction between PET nanoplastics and model organisms constituting freshwater zooplankton. TEM and AFM showed that two-stage ball milling in an aqueous environment allows us to obtain nanometer-scale particles in a relatively simple way. Importantly, the nanoparticles prepared in this way have a variety of shapes, which should correspond well to the irregular morphology of PET nanoplastics formed in actual processes of mechanical degradation of PET waste in natural water bodies. In addition, PET nanoparticles have a strong tendency to stick together and form aggregates of large size, which may facilitate their uptake by filtrating organisms such as *D. magna*.

Our preliminary ingestion experiments confirmed that PET nanoplastics are efficiently taken up by both crustaceans and localize along the entire digestive tract or on the appendages of the individuals. However, confocal microscopy imaging indicates the influence of crustacean type on interactions with PET-M/NPs. In contrast to *D. magna*, the strong adsorption of PET nanoparticles on the surface of the organisms was observed in the case of *T. platyurus* larvae. Acute toxicity tests have shown that despite effective ingestion, the exposure of *D. magna* and *T. platyurus* to PET-M/NPs even at very high concentrations of nanoparticles does not affect the viability of both types of crustaceans. However, even if PET-M/NPs do not show toxicity to organisms after 24 or 48 h, filling the intestine with a large amount of plastic particles can negatively affect food intake in the long term, so further research should focus on long-term toxicity. In conclusion, PET micro/nanoparticles are not toxic to standard test species in freshwater ecosystems at environmentally relevant concentrations or even at very high concentrations with exposure periods of several days.

## Figures and Tables

**Table 1:** Concentration of PET-M/NPs used in toxicity assays.

Concentration ^a^ [mg/L]	*T. platyurus*		*D. magna*	
	# of Individuals ^b^	24 h Survivability [%]	# of Individuals ^b^	48 h Survivability [%]
1000	27	100%	19	100%
100	28	100%	18	100%
10	27	100%	19	100%
1	27	100%	17	100%
0.1	29	100%	13	100%
Control	18	100%	18	94.4%

^a^ The standard freshwater was used as a control and to prepare the PET-M/NP dispersions (see Section 2.6 for details). ^b^ The number of crustacean individuals used in all repetitions.

## Data Availability

Data are contained within the article or Supplementary Materials; further inquiries can be directed to the corresponding authors.

## Supplementary Materials

The following supporting information can be downloaded at https://www.mdpi.com/article/10.3390/polym17091256/s1. Figure S1: Chemical structure of poly(ethylene terephthalate) (PET); Figure S2: Confocal micrographs showing individuals of *D. magna* (A) and *T. platyurus* (B) exposed to a 100 mg/L dispersion of fluorescently labeled (Nile red) PET micro/nanoplastics for 24 (*T. platyurus*) and 48 (*D. magna*) hours; Figure S3: Histogram of the object sizes obtained by grain analysis of the AFM images of the PET particles prepared by the ball milling. The data were used to calculate the average particle size which was 196 ± 94 nm.
