# Supplementary material for: Effect of PET Micro/Nanoplastics on Model Freshwater Zooplankton
Source: Polymers (Basel). 2025 May 5;17(9):1256. doi: 10.3390/polym17091256 (PMC12073589; doi:10.3390/polym17091256)
Supplement: Supplementary file 1 [file polymers-17-01256-s001.zip › polymers-3582127-supplementary.pdf]

polymers

Supplementary Materials

Effect of PET Micro/Nanoplastics on Model Freshwater Zooplankton

Natan Rajtar [1,2], Małgorzata Starek [3], Lorenzo Vincenti [4,5], Monika Dąbrowska [3], Marek Romek [6], Rosaria Rinaldi [4,5], Francesca Lionetto [7,*] and Mariusz Kepczynski [1,*]

[1] Faculty of Chemistry, Jagiellonian University, Gronostajowa 2, 30-387 Kraków, Poland; natan.rajtar@doctoral.uj.edu.pl
[2] Doctoral School of Exact and Natural Sciences, Jagiellonian University, Prof. S. Łojasiewicza 11, 30-348 Krakow, Poland
[3] Department of Inorganic Chemistry and Pharmaceutical Analytics, Faculty of Pharmacy, Jagiellonian University Medical College, 9 Medyczna St, 30-688 Kraków, Poland; m.starek@uj.edu.pl (M.S.); monika.1.dabrowska@uj.edu.pl (M.D.)
[4] Department of Mathematics and Physics "Ennio De Giorgi", University of Salento, Via Monteroni, 73100 Lecce, Italy; lorenzo.vincenti@unisalento.it (L.V.); ross.rinaldi@unisalento.it (R.R.)
[5] Institute for Microelectronics and Microsystems (IMM), CNR, Via Monteroni, 73100 Lecce, Italy
[6] Department of Cell Biology and Imaging, Institute of Zoology and Biomedical Research, Jagiellonian University, 9 Gronostajowa Street, 30-387 Kraków, Poland; marek.romek@uj.edu.pl
[7] Department of Engineering for Innovation, University of Salento, Via Arnesano, 73100 Lecce, Italy
* Correspondence: francesca.lionetto@unisalento.it (F.L.); m.kepczynski@uj.edu.pl (M.K.); Tel.: +39-0832297246 (F.L.); +48-12-686-2532 (M.K.)

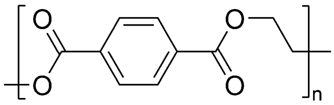

Figure S1. Chemical structure of poly(ethylene terephthalate) (PET).

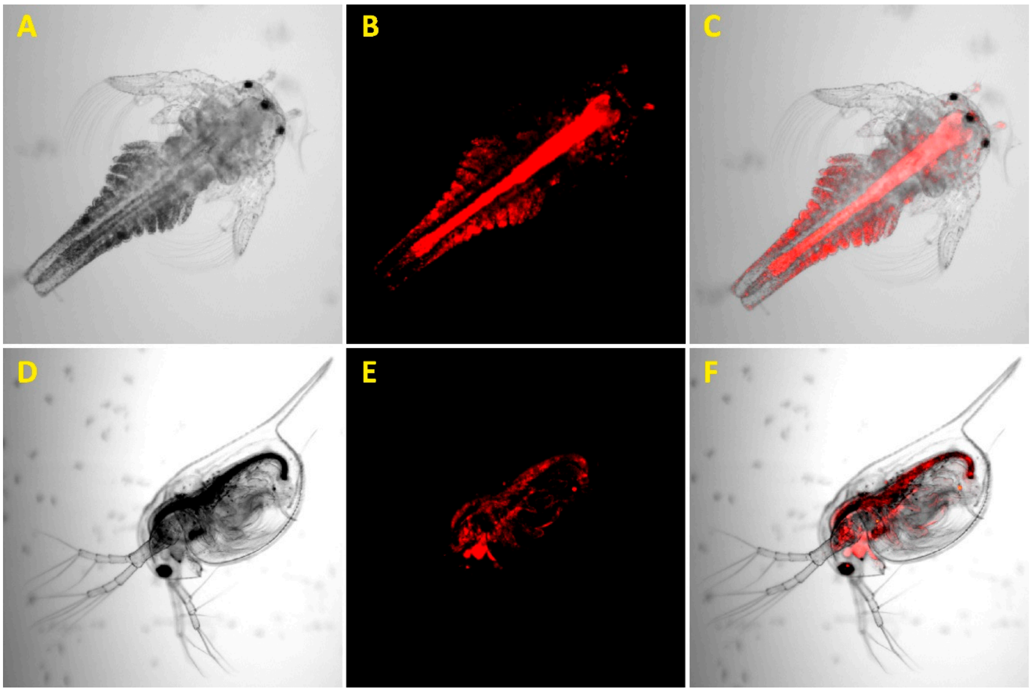

 https://doi.org/10.3390/polym17091256

Figure S2. Confocal micrographs showing individuals of *D. magna* (A) and *T. platyurus* (B) exposed to a 100 mg/L dispersion of fluorescently labeled (Nile red) PET micro/nanoplastics for 24 (*T. platyurus*) and 48 (*D. magna*) hours. Fluorescence from M/NPs (red) was superimposed on the transmitted light images of the crustaceans. The size of *D. magna* and *T. platyurus* individuals is 1.8 and1.4 mm, respectively.

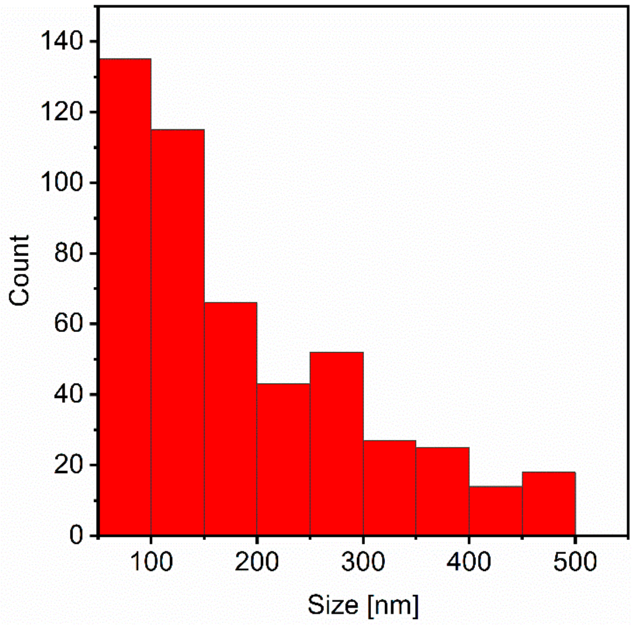

Figure S3. Histogram of the object sizes obtained by grain analysis of the AFM images of the PET particles prepared by the ball milling. The data were used to calculate the average particle size which was 196 ± 94 nm.
